# Niche-related outcomes after caesarean section and quality of life: a focus group study and review of literature

**DOI:** 10.1007/s11136-019-02376-6

**Published:** 2019-12-16

**Authors:** Sanne I. Stegwee, Astrid Beij, Robert A. de Leeuw, Lidwine B. Mokkink, Lucet F. van der Voet, Judith A. F. Huirne

**Affiliations:** 1 Department of Obstetrics and Gynaecology, Amsterdam Reproduction and Development Research Institute, Amsterdam UMC, Vrije Universiteit Amsterdam, De Boelelaan 1117, 1081 HV Amsterdam, The Netherlands; 2 Department of Epidemiology and Biostatistics, Amsterdam Public Health Research Institute, Amsterdam UMC, Vrije Universiteit Amsterdam, De Boelelaan 1089a, 1081 HV Amsterdam, The Netherlands; 3 Department of Obstetrics and Gynaecology, Deventer Hospital, Nico Bolkesteinlaan 75, 7416 SE Deventer, The Netherlands

**Keywords:** Caesarean section, Niche, Quality of life, Chronic disease, Focus groups

## Abstract

**Background:**

A niche in the uterus, present in 60% of women after caesarean section (CS), is associated with several gynaecological symptoms and possibly with subfertility. Studies that focus on quality of life (QoL) in relation to a niche are lacking.

**Purpose:**

To identify niche-related outcomes that influence QoL and to compare patient-reported outcomes with outcomes studied in the literature.

**Methods:**

Two focus group discussions (FGDs, *N* = 8 and 5) were conducted in Amsterdam UMC—location VUmc. Participants were Dutch patients with a large niche, with (planned) surgical treatment for their symptoms. Niche-related symptoms and impact on functioning or participation were fixed topics. The transcripts of the FGDs were coded into outcomes, themes and domains of QoL according to the WHOQOL model. Additionally, participants created a top five important outcomes. Next, we performed a systematic review (SR) on niche-related outcomes and compared the FGDs with niche-related outcomes from the SR.

**Results:**

In four domains (physical health, psychological domain, social relationships and environment), fifteen themes were reported in the FGDs. Abnormal uterine bleeding (AUB), subfertility, sexual activity, abdominal pain and self-esteem were themes prioritised by participants. In the literature, gynaecological symptoms and reproductive outcomes were predominantly studied. Sexuality and self-esteem were prioritised in the FGDs but hardly or never studied in the literature.

**Conclusion:**

We found a broad range of niche-related outcomes influencing QoL. Apart from symptoms evaluated in the literature such as AUB, abdominal pain and subfertility, clinicians and researchers should be more aware of sexual activity and self-esteem in this population.

**Electronic supplementary material:**

The online version of this article (10.1007/s11136-019-02376-6) contains supplementary material, which is available to authorized users.

## Introduction

Caesarean section (CS) rates have increased worldwide over the past decades from 6.7 to 19.1%, with a current European CS rate of 25% of all births [1]. CS is considered to be a safe procedure that can be life saving for both mother and child but the increasing trend draws more attention to adverse outcomes related to CSs [2]. A relatively new long-term sequela is the niche in the uterine caesarean scar. A niche is defined as “*an indentation at the site of the uterine caesarean scar with a depth of at least 2 mm on ultrasound*” [3] and is present in 56–84% of women after one or more CSs [4].

Presence of a niche is associated with multiple symptoms: abnormal uterine bleeding (AUB), e.g. prolonged menstruation and postmenstrual spotting, is present in 30% of women with a niche [4]. Other symptoms include dysmenorrhoea and chronic pelvic pain [5]. Furthermore, the retention of blood, mucus and fluid in the niche, cervix and uterus are hypothesized to be a cause of secondary subfertility, due to unsuccessful sperm cell penetration or embryo implantation [6].

Various therapies have been implemented to treat niche-related problems. For example, both laparoscopic and hysteroscopic niche resection reduce postmenstrual spotting with high satisfaction rates [7–9]. Although niche-related symptoms are not life-threatening consequences after CS, they can cause long-term morbidity and might therefore have serious impact on quality of life (QoL).

The World Health Organisation (WHO) defines QoL as “*an individual’s perception of their position in life in the context of the culture and value systems in which they live and in relation to their goals, expectations, standards and concerns*” [10]. QoL encompasses the domains family, work, environment and health-related quality of life (HRQoL), the latter being the extent to which a medical condition affects someone’s well-being [11] and is used to study the impact of disease or (cost-)effectiveness of interventions. The WHO proposed a conceptual framework resulting in a multidimensional model of four domains containing various ‘facets’, ultimately leading to the assessment of an individual’s QoL (WHOQOL-BREF model) [10].

This concept has not yet been studied substantially in a niche population. Hence, information about factors influencing QoL in this population is incomplete. Qualitative research might gain better insight into the range of symptoms in combination with functioning or participation, by asking open questions and discussing the multilevel character of the condition [12]. With a complete profile we could evaluate in subsequent studies if generic QoL-instruments can accurately measure QoL in this population or whether a disease-specific QoL questionnaire, like the UFS-QoL for leiomyomata, should be designed [13].

We aimed to identify niche-related outcomes that influence QoL in niche patients and, in addition, to compare them to niche-related outcomes reported in the literature, to find discrepancies and similarities.

## Methods

### Focus group discussions

We executed a qualitative study in niche patients to identify outcomes influencing QoL. Outcomes could be a broad range of problems identified as important for patients, such as specific physical symptoms, influence on psychological functioning or social relationships, and environmental factors, as conceptualised in the WHOQOL-BREF model (Online Resource 1) [10]. With this model and our population in mind, we defined QoL as “*an individual’s perception of physical and mental functioning and social participation, including sexual well-being, in relation to symptoms*” [10, 14].

We organised focus group discussions (FGDs) to gain better insight into these patient-reported outcomes that influence QoL, since asking open questions and creating a discussion was crucial to fully understand the range of symptoms in combination with functioning and participation [12, 15]. Compared to one-to-one interviews, FDGs allow for a loose and interactive communication in which participants can complement each other on the different topics without firm guidance of a moderator [12]. Approval of this study was granted by the institutional review board of Amsterdam UMC – location VUmc (2017.362). We completed the checklist of consolidated criteria for reporting qualitative research (COREQ) [16].

#### Inclusion criteria

We organised FGDs among women with a large niche (residual myometrium overlying the niche ≤ 3.0 mm) [17] regardless of wish to conceive. All niches were determined by transvaginal ultrasound and gel instillation sonohysterography. Some patients had already received surgical treatment (hysteroscopic or laparoscopic niche resection, total laparoscopic hysterectomy) for their symptoms or surgery was scheduled. The group size was set at five to ten participants, to create a confidential ambiance for sensitive subject matter but to receive enough input [15, 18].

#### Patient selection

Niche patients were purposively selected and contacted by telephone (A.B.) through the gynaecological outpatient clinic of Amsterdam UMC – location VUmc. Eligible patients had full comprehension of the Dutch language, a maximum travel time of 1 hour and were available on the focus group time and date. Women that volunteered to participate signed the informed consent form before the start of the focus group, which included permission of recording the session for further analysis. Anonymity and confidentiality were ensured. Participants received compensation for their travel expenses.

#### Execution of discussions

The FGDs were held in September 2017 in a VUmc conference room and planned to last approximately 2 hours, following an interview schedule that was prepared in advance. An experienced facilitator (M.B.) led the focus group by allocating speaking turns, meeting the time schedule and asking for further elaboration when information was incomplete. Furthermore, an observer (S.S) and secretary (A.B.) were present for a better overview and interaction with the group and facilitator. No medical relationship was present between any of the participants and the facilitator, observer or secretary. Detailed information is provided in Online Resource 2. Data saturation was expected to be achieved when no new outcomes could be extracted, all items were discussed extensively and further coding was not feasible [19].

The FGDs consisted of multiple steps, in line with previous FGDs organised by members of our research team [20]:

1. Introduction: we explained that this study is performed to gain more insight in relevant outcomes associated with a niche from a patient's perspective. Participants gave an overview of the history of their disease and their motivation to participate in the study.
2. Post-it session: participants discussed the problems (outcomes) they experienced on physical, psychological, social or environmental level and how this affected their QoL. Each participant was asked to write down two problems on post-its. The group collectively discussed the problems, which were visualised on a flip over board, and when there was no further input, a pre-set list comprising outcomes from clinical practice and previous research [4] was checked for relevance (see Online Resource 3).
3. Prioritisation: the importance of niche-related outcomes concerning QoL was established by asking the participants to prioritise all the outcomes that were mentioned in the discussion in a written top five list without further discussion with other participants. The listed outcomes were appointed one to five points, with number one being allocated five points, number two being allocated four points et cetera. This resulted in a total relevance score for all outcomes, which could be summarised into a general top five list ranked according to importance. Furthermore, they were asked to indicate which domain (physical, psychological, social, environmental) was experienced most limiting.

#### Data analysis

The FGDs were transcribed verbatim with the recordings and analysed in Atlas.ti. A template analytic approach with inductive components was used for the data analysis [21, 22]. After getting familiar with the data, one reviewer (A.B.), guided by an experienced researcher (S.S.), made a coding tree with relevant codes for the research purpose. During the first coding round, the specific codes were applied to quotes of participants. Similar codes were merged and categorised into outcomes. One level higher, we created themes that were labelled after the ‘facets’ of the WHOQOL framework if feasible, in which the outcomes fitted. These themes were subsequently incorporated in the predefined WHOQOL domains: (1) physical health, (2) psychological domain, (3) social relationships, (4) environment (see Online Resource 1) [10]. Then, the coding process was evaluated on completeness and right categorisation by a project supervisor (J.H.). The final codes were entered into a table, which summarises the data and gives an overview of associations. Since the FGDs were held in Dutch, quotes were translated to English for the results table.

### Systematic review of literature

Additionally, to supplement a part of a previously performed systematic review [4] with recently published niche-related outcomes, we repeated the search. We searched PubMed and Embase.com (S.S. and A.B.) from February 2013, when the previous search was performed, up to June 2019. The complete search strategy can be found in Online Resource 4. We have focused on symptoms related to niches or thin lower uterine segment to determine the range of outcomes reported in the literature, either measured by clinicians or reported by patients.

We included full-text published randomised controlled trials, cohort and case–control studies written in the English language. Articles written in other languages and case reports, case series and systematic reviews were excluded. Studies were selected according to the PRISMA guidelines [23]. We included studies when they reported on patients with at least one previous CS and/or a niche, in whom the scar was evaluated with ultrasound. Included studies could either report on 1) outcomes in a random population after CS or on 2) outcomes in asymptomatic and symptomatic patients with a niche confirmed by TVUS or on 3) outcomes after medicinal or surgical therapy in symptomatic niche patients.

First, S.S. and A.B. screened titles and abstracts of the records independently. Subsequently, the same reviewers independently assessed full-text papers of possibly eligible articles after the first screening. Any disagreement in the screening and assessment of the papers was dissolved through discussion between the two reviewers, and if necessary discussed with a third reviewer (J.H.).

We extracted data on study characteristics and design, type of participants (asymptomatic or symptomatic, with or without treatment), evaluation with transabdominal or transvaginal ultrasound with or without the use of contrast, intervention and control group (if applicable) and primary and secondary outcome measures. No quality assessment was performed due to the narrative character of this review.

## Results

### Focus group discussions

Two FGDs were conducted among thirteen niche patients, including eight and five women, respectively. The first FGD lasted 1 h and 57 min and the second 2 h and 18 min. The second FGD supplemented the outcomes mentioned in the first FGD, but no essential outcomes were added, so data saturation was considered to be achieved. Table 1 presents the participants’ characteristics. In the transcripts, 80 open codes were identified, which were merged into the fifteen themes with related outcomes during axial coding. This led to the three-level dimension of QOL features in our FGDs: outcome, theme and domain. Table 2 shows a representation of the domains with themes and outcomes raised, accompanied by illustrative quotations from the discussion which are further explained below. Additional information is provided in Online Resource 4.

**Table 1:** Focus group discussion participants’ characteristics

Characteristic	Value
Age, median (range)	38 (30-42)
Number of children, mean	2.2
Number of caesarean sections, mean	1.8
Active wish to conceive	5 (38)
Years since diagnosis	
< 2 years	4 (31)
2–4 years	4 (31)
> 4 years	5 (38)
Currently pregnant	1 (8)
Received niche therapy	
Laparoscopic niche resection*	6 (46)
First laparoscopic, then hysteroscopic niche resection	3 (23)
Total laparoscopic hysterectomy	3 (23)
Fertility treatment	
Fertility treatment due to niche	5 (38)
Fertility treatment due to previous infertility	0 (0)

N = 13

Values are the number of participants with (%) unless otherwise indicated

*Performed under hysteroscopic guidance

**Table 2:** Results from focus group discussions: the four domains with themes, outcomes and illustrative quotations

Theme	Outcome	Quote
*Physical health*		
Abnormal uterine bleeding	Intensity (volume and duration) of AUB	“(…), like I had during the holidays, early in the morning. I just sat on the toilet for fifteen minutes because it kept gushing.”
	Irregularity of AUB	“It just starts at an inappropriate time, so after intercourse or when you’re just about to go to your work or ready to leave the house. And then, you’re covered in blood again.”
	Odour due to AUB	“I was mainly wondering: can’t everybody smell me?”
Abdominal pain	(Chronic) abdominal pain	“Out of the 30 days, there might have been two days that I thought: oh, my belly doesn’t hurt for a change.”
	Caesarean scar sensations	“Looking back, I felt the scar… I felt a bad pulling sensation in my scar, but I thought it was just part of the recovery.”
Subfertility	Inability to conceive	“It doesn’t matter if it’s the first, second or third. When you’re not able to conceive anymore, that’s very intense.”
	Pregnancy anxieties	“It was my biggest fear to get contractions *(due to thin myometrium)*.”
	Negative advice	“Yes, that’s no longer there *(wish to conceive)*. Well, maybe there was, but not anymore. No, I’m not allowed *(to get pregnant)* anymore.”
Urological symptoms	Polyuria	“I have to pee more often at night.”
	Painful micturition	“I feel pain during micturition or when my bladder is full.”
Energy and fatigue	Not specified	“If you’re dealing with the pain all day and you have so much blood loss; that costs a lot of energy.”
Activities of daily living	Not specified	“(…) such heavy pain that you just need to lie in bed with a paracetamol all day *(during menses)*.”
Work capacity	Period related	“I’m not going to say (*to her employer*): ‘I can’t come to work because I am having my period’” and “It’s the first two days that I bleed that heavily. I don’t actually leave the house then *(to go to work)*, because if I do - I did it sometimes - I have to go and change pads every half an hour or more.”
	Pregnancy related	“At 20 weeks, the doctor said: ‘your myometrium is so thin…’ So I stopped working at the gestational age of 24 weeks.”
	In general	“I had a job in education, and it is just not possible to combine this with a hospital life *(scheduled niche appointments and fertility treatments)*” and “I informed my superiors: I can’t stand all day, so we have to make a plan together.”
*Psychological domain*		
Self-esteem	Self-image	“Because I had bleedings for so long, yeah, you don’t really get clean and fresh anymore. You’re kind of disgusted by yourself.”
	Self-doubt	“Am I crazy or is there really something going on?”
Preoccupation	Not specified	“Just going a day to the beach and thinking: Oh, do I have pads with me or where is the nearest bathroom? It’s just always on your mind.“
Negative feelings	Cause/blame	“Should I have done things differently? Where did it go wrong? With the CS or the IUD? Or wouldn’t it have gotten so bad, if I had reached out earlier?”
	Loneliness or depression	“The limitations in your daily life and the loneliness in this compared to your age group.”
*Social relationships*		
Social support	Social acceptance	“You don’t talk about it. Who’s going to talk about periods, when you’re spending the night with friends?”
	Unfamiliarity	“A lot of people don’t know what it is *(a niche)*.”
Personal relationships	Being heard	“People just don’t take you seriously when you suffer from the effects *(of CS)* and 99% of women does not.”
	Family life	“And there *(at work)* I have to pull myself together, but at home… When I’m free and the little one is in bed, then I’m done. I would do nothing.”
	Incomprehension	“That’s the worst part, that my partner is okay with it. And that’s very sweet, you know, but I’m not. It’s very annoying, because it’s almost impossible for them to understand.”
Sexual activity	Embarrassment	“Checking during intercourse; am I bleeding now? That’s nasty.”
	Meet expectations	“That was the worst part for me. Of course, you’re bleeding, but you have a partner that also wants something.”
	Dyspareunia	“If you’re thinking: oh, it’s going to hurt, it’s going to hurt *(the intercourse)*… I don’t even want it anymore, because it hurts and to get over that…”
	Avoiding intercourse	“My sex life is on a very low level right now.”
*Environment*		
Healthcare system	Knowledge	“Four years ago, it was all unknown as well. Why doesn’t every general practitioner know about this by now? That’s strange, right?”
	Acknowledgement	“You’re telling your story to professionals and yet those professionals don’t really hear you. You’re being sent from pillar to post.”
	Lack of treatment options	“The general practitioner shouldn’t set you up with contraception pills for six times and only afterwards consider sending you to a gynaecologist.”
Participation in leisure activities	Not participating in leisure activities	“In those five days *(free of bleeding)*, you are not going to the gym. You’re just happy to be free and clear.”
	Discomfort during leisure activities	“When I went to the spa, I was like: let’s take a dark towel with me, because if I take a white one and something happens… *(irregular blood loss).”*

AUB: abnormal uterine bleeding, CS: caesarean section, IUD: intra-uterine device

#### Physical health

In this domain, all physical outcomes in relation to the niche were discussed. AUB comprises both spotting and the menstrual period. The volume and duration of AUB, referred to as ‘intensity’ in Table 2, and (chronic) abdominal pain were identified as limiting symptoms for daily life activities and work capacity. Women felt they had to be in proximity to a bathroom and be able to go there frequently. Since intensity of AUB and abdominal pain were mainly related to the menstruation, women were mostly restricted in this period of time. Some women preferred staying at home—and working home if possible—the first days of their period. Limited working capacity in general was exceptional, although one participant could not combine the inconvenience of hospital visitations and surgical interventions with her job and was forced to resign.

Furthermore, the outcomes ‘irregularity of AUB’ and ‘AUB-associated odour’ were identified. The irregularity of AUB covers the unpredictability of the onset of bleeding. Mainly sexual activity but also lifting (e.g. a younger child) could suddenly cause heavy bleedings. AUB-associated odour was described as an embarrassing sensation that caused distress over the cognizance of others.

The inability or the proceedings required to conceive again (i.e. subfertility) had an immense impact on QoL, also for women who ‘chose’ to have a total laparoscopic hysterectomy. Four women had been involved in unsuccessful fertility treatments, one was still engaged in the process. Furthermore, some women diagnosed with a niche described their following pregnancy as displeasing and stressful, due to concerns regarding their thin residual myometrium and possible risk for uterine rupture.

The other physical themes, urological symptoms and sensations in the caesarean scar, were part of the physical symptoms that influence functioning and QoL but were regarded as less obstructing than those described above. Urological symptoms were only recognised as niche-associated after the outcomes were introduced by the moderator from the pre-set list.

#### Psychological domain

The theme ‘self-esteem’ in the psychological domain is covered by personal insecurities and bodily image, and was related to AUB: “*Those pants, you don’t feel fresh, you don’t feel good, you don’t feel like wearing something nice*.”. One participant experienced unexpected AUB as an obstacle in her new relationship: “*Certainly when you just have a new partner. You already feel insecure. Like ‘maybe we can dim the light?*’”. Personal insecurities could also derive from health professionals’ lack of knowledge, which caused self-doubt about the severity of their symptoms. One of the patients commented about her doctor: “*He said: ‘the complaints that you’re talking about, I have never heard of that before*.'”.

Daily preoccupation with niche-related symptoms was repeatedly mentioned in the discussions. Sometimes, it involved daily actions: Moderator: “*The ones who experienced odour, what did you do about that*?”. R1: “*Change as frequent as possible*.”, R2: “*Change often, washing, flushing*…”. However, the preoccupation was mainly psychological and included concerns on how their daily schedule could be combined with their medical condition. This required proper organization and was experienced as energy consuming.

#### Social relationships

Participants experienced difficulties in communicating about their condition with others. Two outcomes regarding ‘social support’ were identified: (1) acceptance: niche-related symptoms are considered to be a taboo, (2) incomprehension: others are unfamiliar with the subject. Personal relationships were also influenced, since patients felt that their partner, family, friends or colleagues were not taking them seriously. Some women managed to do their work and daily life activities but were exhausted when they got home, which then affected their family life.

Furthermore, sexual activity was very important for the QoL of niche patients. Sexual problems, such as decreased libido or lubrication, or aversion to sexual activity could arise due to bleedings or dyspareunia. This was often accompanied with a fear of disappointing their partner and a sense of embarrassment due to a lowered self-esteem. As this participant states: “*Unfortunately, those bleedings hinder me, if you want to talk about limitations really quick: it’s not very good for your sex life, a lot of Candidiasis, fungal infections, because you have those bleedings all the time and you have to wear pads*.”. Problems with subfertility, an outcome shown within the physical domain, could further enlarge frustrations concerning sexual activity: “*It’s necessary to time intercourse during ovulation, however given the bleeding until the ovulation I was trapped into a very difficult situation*.”.

#### Environmental factors

Outcomes that belong to ‘healthcare system' were relevant for QoL. The unfamiliarity of many care providers with a niche caused frustrations, such as delayed referral to a gynaecologist or numerous contacts and physical examinations before diagnosis. The time from niche complaints after CS to the diagnosis was considered unnecessarily long and most women were sent home multiple times because the reported complaints were considered as part of normal recovery after CS.

Furthermore, participation in leisure activities was compromised. “*You don’t go swimming or to the spa. It’s just very unpleasant*.”. When patients were engaged in leisure activities, they could feel uncomfortable or distressed because symptoms might occur suddenly.

#### Prioritisation

Although all outcomes could be reported, participants only prioritised themes in their top five. The overall top five was derived from the relevance scores of both FGDs:

1. Abnormal uterine bleeding
2. Subfertility
3. Sexual activity
4. Abdominal pain
5. Self-esteem

**Fig. 1:**
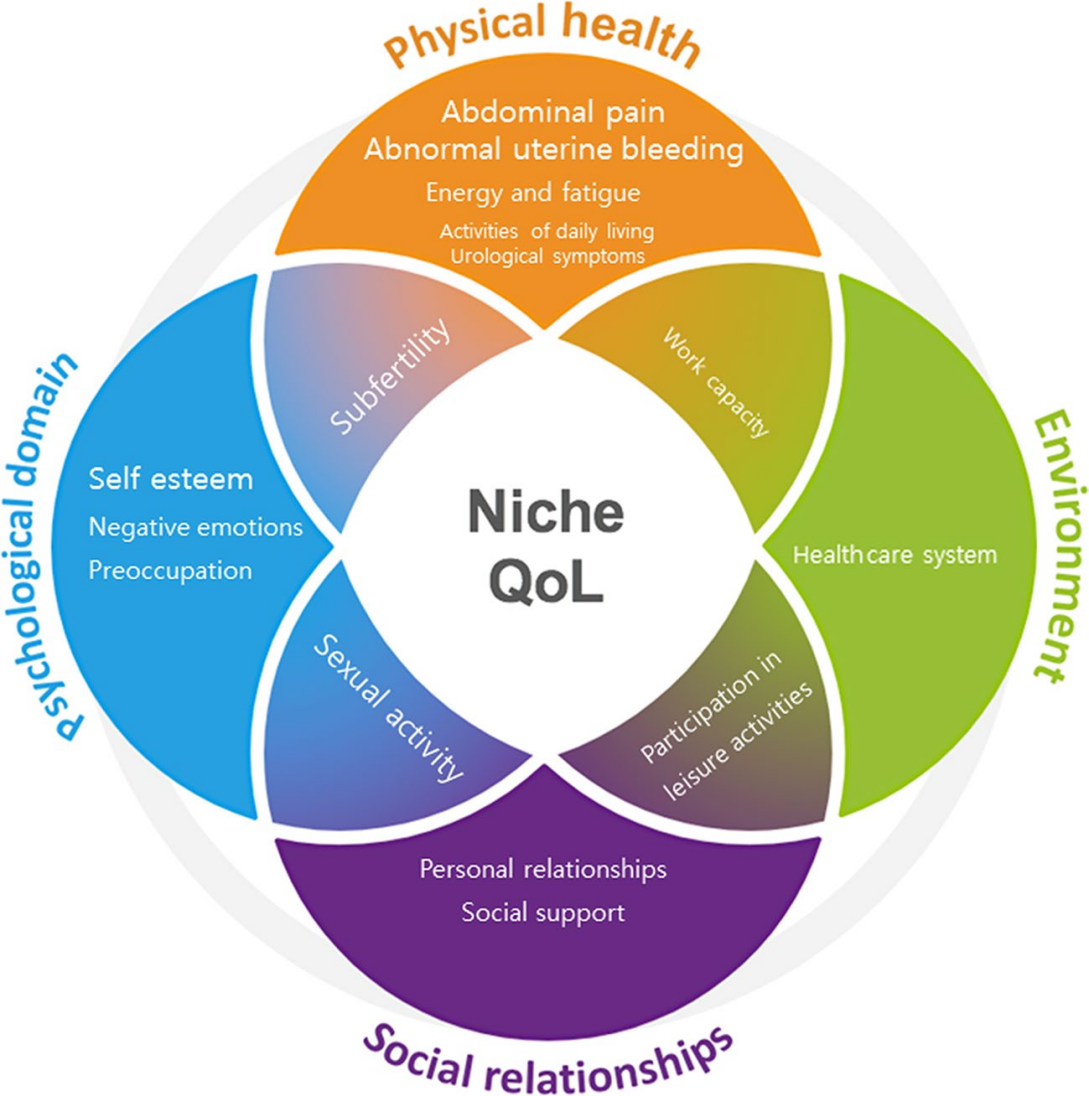
Connection of domains and themes reported by niche patients. Size indicates relevance of the theme for QoL, with larger themes being reported more frequently or prioritised in the focus groups

Other top five reported outcomes were AUB-associated odour, polyuria, energy and fatigue, personal relationships, pregnancy anxiety, psychological complaints and healthcare system features. The total relevance score of these candidate outcomes was not high enough to reach the overall top five. Furthermore, we tried to establish what domain was most limiting for the participants, but they all (N = 13) stated that the complete profile of their condition, including all reported themes and domains influenced their QoL. The fifteen themes and connectedness of domains are visualised in Fig. 1, with larger displayed size indicating relevance.

### Systematic review of literature

The complete reviewing process and the results of the literature search can be found in Online Resource 5. We included 39 articles that were published after February 2013. Bij de Vaate et al. identified seven articles on the same topic evaluating the scar with ultrasound, published until February 2013 [4]. All 46 articles including explanatory and summarised information are presented in Online Resource 6. Reported outcomes, either by patients or clinicians, are summarised in Table 3. The most frequently reported gynaecological outcomes were prolonged menstrual bleeding [24–43], postmenstrual spotting [5, 8, 9, 25, 35, 44–50], dysmenorrhoea [5, 8, 9, 25, 27], dyspareunia [5, 27, 46] and (chronic) pelvic pain or abdominal pain [5, 24, 25, 27, 34, 40, 46, 51, 52]. Prevalence of abnormal adhesive placenta [26, 27], successful vaginal birth after CS [27, 53–55] and uterine rupture or dehiscence [27, 56–63] were reported as obstetric outcomes. Fertility-related niche therapy studies reported on wish to conceive (secondary subfertility) [26, 27, 33, 52], pregnancy [25, 26, 33, 39, 40, 42, 64] and miscarriage rate [26, 27, 33, 39, 42, 43], the risk of caesarean scar pregnancy [27, 33, 40] and live birth rate [26, 27, 40, 43]. Subsequent pregnancies were followed after niche therapy in four studies: mode and timing of delivery [39, 42], risk of abnormal adhesive placenta [40] and risk of uterine dehiscence or rupture [39, 40, 43] were reported. Three publications reported on change in HRQoL, assessed using the validated SF-36 questionnaire, after niche therapy (surgical or hormonal) [8, 9, 24]. One study evaluated sexual functioning after therapy versus expectant management [9].

**Table 3:** Summary of outcomes reported in the literature in relation to a niche or thin lower uterine segment

**Gynaecological symptoms in niche population**	N = 11
Bleeding abnormalities	N = 10
Prolonged menses [25, 35]	2
Postmenstrual spotting [5, 25, 35, 44, 45, 47–50]	9
Abnormal uterine bleeding (not further specified) [70]	1
Pain	N = 2
Dysmenorrhoea [5, 25]	2
Chronic pelvic pain [5, 25]	2
Dyspareunia [5]	1
Other	N = 2
Risk on failed early TOP [71]	1
Urinary incontinence [44]	1
**Gynaecological symptoms after therapy (surgical or hormonal)**	N = 24
Bleeding abnormalities	N = 24
Prolonged menses [24, 26–34, 36–39, 41–43]	17
Postmenstrual spotting [8, 9, 46]	3
(Postmenstrual) abnormal uterine bleeding [40, 51, 52, 72]	4
Pain	N = 10
Dysmenorrhoea [8, 9, 27]	3
Abdominal pain [24, 27, 34, 52]	4
Dyspareunia [27, 46]	2
Pain during micturition [9]	1
Pelvic pain [40, 46, 51]	2
**Reproductive outcomes in population with niche or thin LUS**	N = 19
Fertility related—after therapy (surgical or hormonal)	N = 8
Wish to conceive [26, 27, 33, 52]	4
Pregnancy rate [25, 26, 33, 39, 40, 42, 43]	7
Miscarriage rate [26, 27, 33, 39, 42, 43]	6
Caesarean scar pregnancy [27, 33, 40]	3
Live birth rate [26, 27, 40, 43]	4
Obstetrics related—after therapy (surgical or hormonal)	N = 4
Mode and/or timing of delivery [39, 42]	2
Risk of abnormal adhesive placenta [40]	1
Risk of uterine dehiscence/rupture [39, 40, 43]	3
Obstetrics related—no treatment	N = 12
Risk of abnormal adhesive placenta [26, 27]	2
Association US and risk of uterine dehiscence/rupture [27, 56–63]	9
Association US and % successful VBAC [27, 53–55]	4
**Evaluation on a functional level after therapy (surgical or hormonal)**	N = 3
Quality of life (SF-36) [8, 9, 24]	3
Sexuality (FSFI) [9]	1

N: number of articles, TOP: termination of pregnancy, LUS: lower uterine segment, US: ultrasound, VBAC: vaginal birth after caesarean, SF-36: Short Form 36-item Health Survey, FSFI: Female Sexual Functioning Index

### Outcomes from FGDs compared with outcomes reported in the literature

**Fig. 2:**
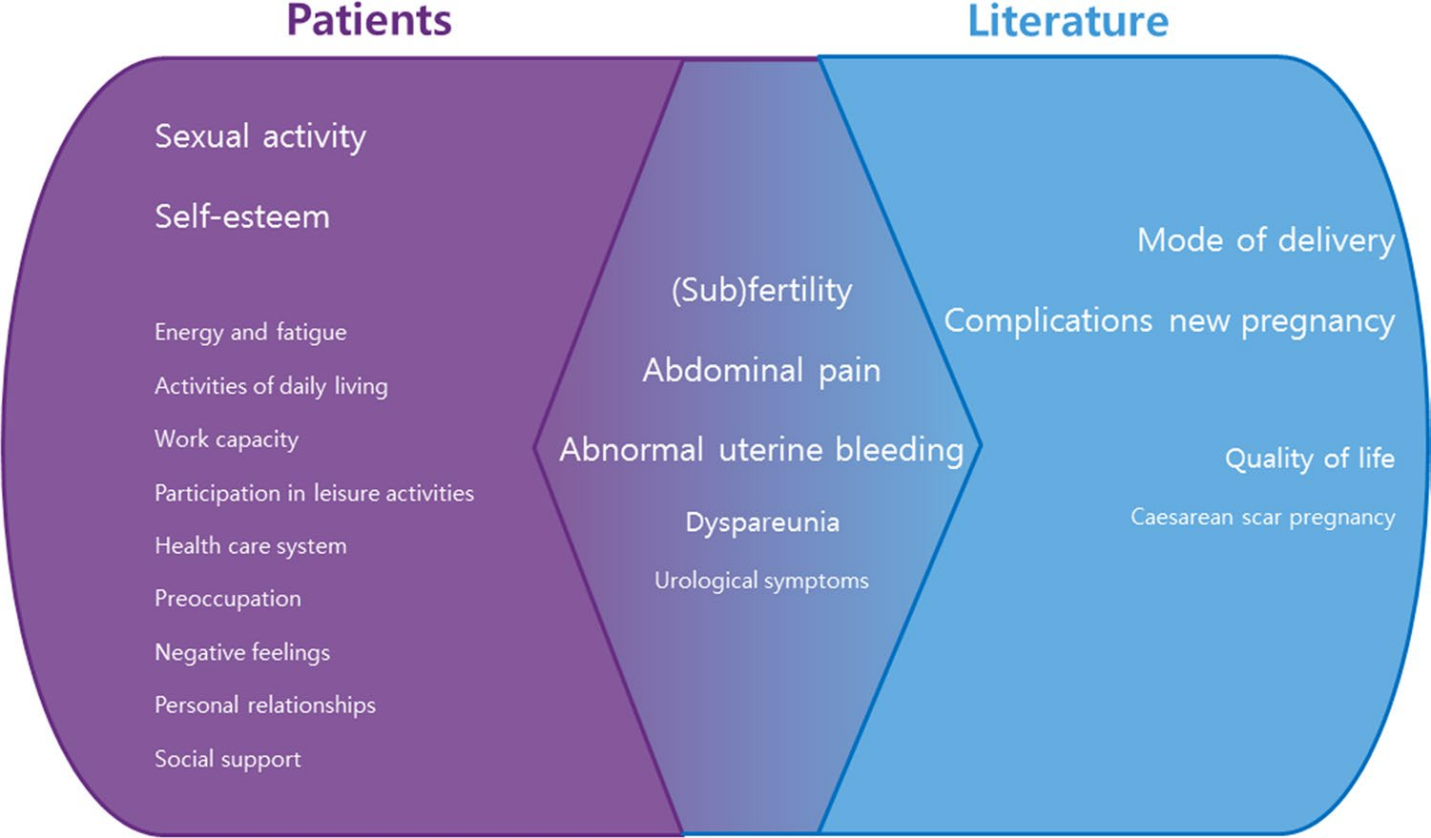
Discrepancies and similarities in themes reported by patients and in the literature. Size indicates relevance in focus group discussions or frequency of reporting in the literature, with larger themes being reported more often

Patient-reported outcomes after prioritisation in our FGDs were AUB, subfertility, sexual activity, abdominal pain and self-esteem. The outcomes that were studied most frequently in the niche literature were gynaecological symptoms (bleeding abnormalities, pain) and reproductive outcomes (fertility outcomes after surgery, obstetrics-related problems). In these two lists, some overlap exists but sexual activity and self-esteem were prioritised by patients and hardly studied in the literature (see Fig. 2).

## Discussion

### Main findings

This study was designed to evaluate outcomes that are important for the QoL of niche patients and to compare these with outcomes reported in the literature. In the prioritised top five AUB, subfertility and abdominal pain from the domain ‘physical health’, self-esteem from the psychological domain and sexual activity from the domain ‘social relationships’ were considered most relevant for QoL by the participants.

Outcomes studied in the literature were mainly gynaecological symptoms reported by patients, and reproductive outcomes. The overlap in reported outcomes by patients and in the literature were in the physical domain. AUB, abdominal pain and subfertility were prioritised by patients and studied most frequently in the literature. Other aspects such as sexual activity and self-esteem are absent in the evaluation of niche-related symptoms according to our systematic review, but were reported relevant in our FGDs.

### Strengths and limitations

To the best of our knowledge, this is the first study that qualitatively as well as systematically investigated the full range of outcomes caused by a niche and in which outcomes studied in the literature and patient-reported outcomes were compared. The FGDs have multiple strengths; there was a respectable sense of confidentiality and the women offered us personal stories and in-depth information about their lives. An experienced moderator was present who ensured complete information and equal participation of all women. Another strength is that we used a pre-designed interview schedule for both FGDs and we used the WHOQOL model for categorization during data analysis. This fitted our data well, as many ‘facets’ of this model were mentioned in our FGDs, which we categorised as ‘themes’. A limitation of this study was that we did not stratify for demographic characteristics such as education level, religion or ethnicity. Furthermore, participants who are more seriously restricted by niche-related symptoms might have shown a higher willingness to participate, although we think that only less and not different outcomes would have been mentioned if we had invited less seriously restricted patients. Another limitation is that we did not write a research protocol, although we did follow our interview schedule for both discussions and we completed the checklist of consolidated criteria for reporting qualitative research.

### Comparison with the previous literature

To our knowledge, no qualitative studies regarding niches were previously published, although the identified themes and outcomes are in line with qualitative studies on AUB. Matteson and Clark developed a QoL model for AUB showing that patients with bleeding-related symptoms and episodic social embarrassment have underlying routines and rituals to prevent embarrassment, such as physical preparations or avoiding social activities. These rituals and routines are probably not adequately covered during clinical assessment [65]. Santer et al. reported pain, heaviness, irregularity and general inconvenience as most bothersome to women with AUB [66]. Mood fluctuations and tiredness in relation to a menstruation reported through their interviews were considered less relevant in our population. Moreover, the SR and meta-ethnography of Garside, Britten and Stein reports that women have different attitudes towards AUB with multiple internal and external barriers for women to define AUB as a problem requiring medical help [67]. Our participants also reported self-doubt concerning the reality of their symptoms, but it did not prevent them from seeking help initially. This could be due to the structure of the Dutch healthcare system or systematic short-term check-up after CS.

We identified no recent systematic reviews about symptoms associated with a niche. Two reviews about treatment of niches were published, in which symptoms were also mentioned [68, 69]. Abnormal uterine bleeding, pelvic pain, infertility, dyspareunia and dysmenorrhoea were reported, which is comparable to our results. It is noticeable that we found 39 articles reporting on symptoms over the last six years, whereas the search of Bij de Vaate et al. revealed eight studies up to February 2013. This difference in number of articles reflects the recent interest in niches, risk factors, symptoms and treatments. Fertility problems in relation to a niche were not reported in the previous review, nor were symptoms after treatment [4], which suggests that this is only studied recently.

### Future perspectives and implications

Outcomes considered relevant for patients in our FGDs could contribute to the evaluation of health instruments to measure QoL in niche patients. Additional research is needed to assess the quality of currently available outcome measurement instruments or to develop new instruments for this specific population. Our study can be used as a base for clinicians for the communication with niche patients. To provide better medical care, further qualitative and quantitative research—preferably on a larger and international scale—is important. A future perspective is to achieve international consensus in creating a core outcome set for niche-related symptoms, involving patients, general practitioners, gynaecologists, and researchers.

Our study underlines the impact of niche-related problems for individual patients, which should be taken seriously by physicians. Therefore, increasing awareness of niche-related problems and the direct relation to a previous CS should be the focus of future healthcare programs in obstetrics and gynaecology as well as for general practitioners.

## Conclusion

The most important themes that influenced QoL in our study population of niche patients were AUB, subfertility, sexual activity, abdominal pain and self-esteem. In the literature, physical symptoms (AUB, subfertility and abdominal pain) and obstetric outcomes measured by clinicians were studied most frequently. Sexual activity and self-esteem were important themes associated with QoL reported by niche patients but rarely or never a topic of interest in the niche literature.

## Electronic supplementary material

Below is the link to the electronic supplementary material.

Supplementary material 1 (DOCX 14 kb)

Supplementary material 2 (DOCX 12 kb)

Supplementary material 3 (DOCX 13 kb)

Supplementary material 4 (DOCX 16 kb)

Supplementary material 5 (TIFF 14141 kb)

Supplementary material 6 (DOCX 33 kb)

## Abbreviations

CS: Caesarean section
AUB: Abnormal uterine bleeding
QoL: Quality of life
SF-36: 36-Item Short Form Health Survey
EQ-5D: EuroQoL-5D
HRQoL: Health-related quality of life
WHO: World Health Organization
FGD(s): Focus group discussion(s)
VUmc: VU University Medical Centre
