# Supplementary material for: Niche-related outcomes after caesarean section and quality of life: a focus group study and review of literature
Source: Qual Life Res. 2019 Dec 16;29(4):1013–25. doi: 10.1007/s11136-019-02376-6 (PMC7142042; doi:10.1007/s11136-019-02376-6)
Supplement: Supplementary file 1 — Supplementary material 1 (DOCX 14 kb) [file 11136_2019_2376_MOESM1_ESM.docx]

Electronic supplementary material – Online Resource 1

| Domain | Facets incorporated within domains |
| --- | --- |
| Physical health | Activities of daily living Dependence on medicinal substances and medical aids  Energy and fatigue  Mobility  Pain and discomfort  Sleep and rest  Work Capacity |
| Psychological | Bodily image and appearance Negative feelings  Positive feelings  Self-esteem  Spirituality/Religion/Personal beliefs  Thinking, learning, memory and concentration |
| Social relationships | Personal relationships Social support  Sexual activity |
| Environment | Financial resources Freedom, physical safety and security  Health and social care: accessibility and quality  Home environment  Opportunities for acquiring new information and skills  Participation in and opportunities for recreation/leisure activities  Physical environment (pollution/noise/traffic/climate)  Transport |

**Online Resource 1.** *WHOQOL-BREF list of domains and facets*
