# Supplementary material for: Niche-related outcomes after caesarean section and quality of life: a focus group study and review of literature
Source: Qual Life Res. 2019 Dec 16;29(4):1013–25. doi: 10.1007/s11136-019-02376-6 (PMC7142042; doi:10.1007/s11136-019-02376-6)
Supplement: Supplementary file 2 — Supplementary material 2 (DOCX 12 kb) [file 11136_2019_2376_MOESM2_ESM.docx]

Electronic supplementary information - Online resource 2

**Additional information from the methods section**

Focus group discussions

Experienced facilitator: M.B., medical doctor, female, no medical relationship with any of the participants

Observer: S.S., medical doctor, female, PhD-student on this topic, no medical relationship with any of the participants

Secretary: A.B., BSc, female, medical student writing thesis about this topic, no medical relationship with any of the participants

**Additional information from the results section**

Focus group discussions

39 eligible women denied participation: without further specification (n=26), due to lack of time (n=4), other diseases that impair mobility (n=4), not able to remember symptoms (n=4) or did not want to share personal information (n=1).

Transcripts were not returned to participants for comments and participants were not asked to provide feedback on the findings.
