# Supplementary material for: Niche-related outcomes after caesarean section and quality of life: a focus group study and review of literature
Source: Qual Life Res. 2019 Dec 16;29(4):1013–25. doi: 10.1007/s11136-019-02376-6 (PMC7142042; doi:10.1007/s11136-019-02376-6)
Supplement: Supplementary file 3 — Supplementary material 3 (DOCX 13 kb) [file 11136_2019_2376_MOESM3_ESM.docx]

Electronic supplementary information – Online Resource 3

| Category | Symptom |
| --- | --- |
| 1. Gynaecological | Prolonged menstruation  Spotting (postmenstrual or intermenstrual)  Dysmenorrhea  Abdominal pain  Pain or sensitivity in (abdominal) caesarean scar  Vaginal discharge |
| 1. Urological | Pain when having a full bladder  Pain after micturition  Frequent micturition |
| 1. Sexual | Contact bleeding  Dyspareunia  Decreased libido |
| 1. Fertility | (in)ability of spontaneous pregnancy  Problems with fertility treatment *(ART)* |

**Online Resource 2.** *Pre-set list of niche-related symptoms derived from clinical perspective and literature. ART: assisted reproductive technology such as intra-uterine insemination (IUI), in vitro fertilisation (IVF) or intracytoplasmatic sperm injection (ICSI).*
