# Supplementary material for: Niche-related outcomes after caesarean section and quality of life: a focus group study and review of literature
Source: Qual Life Res. 2019 Dec 16;29(4):1013–25. doi: 10.1007/s11136-019-02376-6 (PMC7142042; doi:10.1007/s11136-019-02376-6)
Supplement: Supplementary file 4 — Supplementary material 4 (DOCX 16 kb) [file 11136_2019_2376_MOESM4_ESM.docx]

Electronic Supplementary material – Online Resource 4

**Online Resource 4: Literature search**

PubMed 21 June 2019

| Search | Query | Results |
| --- | --- | --- |
|  | Date range 2013-02-01 to 2019-06-21 | 887 |
| #4 | #1 AND #2 AND #3 | 2313 |
| #3 | "Cesarean Section"[Mesh] OR cesarea*[tiab] OR caesarea*[tiab] OR “c section”[tiab] OR “c sections”[tiab] OR (abdominal[tiab] AND deliver*[tiab]) OR postcesarea*[tiab] OR postcaesaria*[tiab] | 76187 |
| #2 | "Uterus"[Mesh] OR "Uterine Diseases"[Mesh] OR uterus[tiab] OR uterine[tiab] OR myometri*[tiab] OR endometri*[tiab] OR endomyometri*[tiab] OR myoendometri*[tiab] | 339156 |
| #1 | "Cicatrix"[Mesh] OR cicatr*[tiab] OR scar[tiab] OR scars[tiab] OR scarring[tiab] OR isthmocele*[tiab] OR niche[tiab] OR niches[tiab] OR anechoic[tiab] OR pouch*[tiab] OR diverticul*[tiab] | 177761 |

Embase 21 June 2019

| Search | Query | Results |
| --- | --- | --- |
|  | Date range 2013-02-01 to 2019-06-21 | 1754 |
| #5 | #1 AND #2 AND #3 AND [embase]/lim | 3019 |
| #4 | #1 AND #2 AND #3 | 3558 |
| #3 | 'cesarean section'/exp OR cesarea*:ab,ti OR caesarea*:ab,ti OR 'c section':ab,ti OR 'c sections':ab,ti OR (abdominal:ab,ti AND deliver*:ab,ti) OR postcesarea*:ab,ti OR postcaesarea*:ab,ti | 121471 |
| #2 | 'uterus'/exp OR 'uterus disease'/exp OR uterus:ab,ti OR uterine:ab,ti OR myometri*:ab,ti OR endometri*:ab,ti OR endomyometri*:ab,ti OR myoendometri*:ab,ti | 458480 |
| #1 | 'wound dehiscence'/exp OR 'scar formation'/exp OR 'scar'/exp OR cicatr*:ab,ti OR scar:ab,ti OR scars:ab,ti OR scarring:ab,ti OR isthmocele*:ab,ti OR niche:ab,ti OR niches:ab,ti OR anechoic:ab,ti OR pouch*:ab,ti OR diverticul*:ab,ti | 245828 |
