# Supplementary figures and images for: Niche-related outcomes after caesarean section and quality of life: a focus group study and review of literature
Source: Qual Life Res. 2019 Dec 16;29(4):1013–25. doi: 10.1007/s11136-019-02376-6 (PMC7142042; doi:10.1007/s11136-019-02376-6)

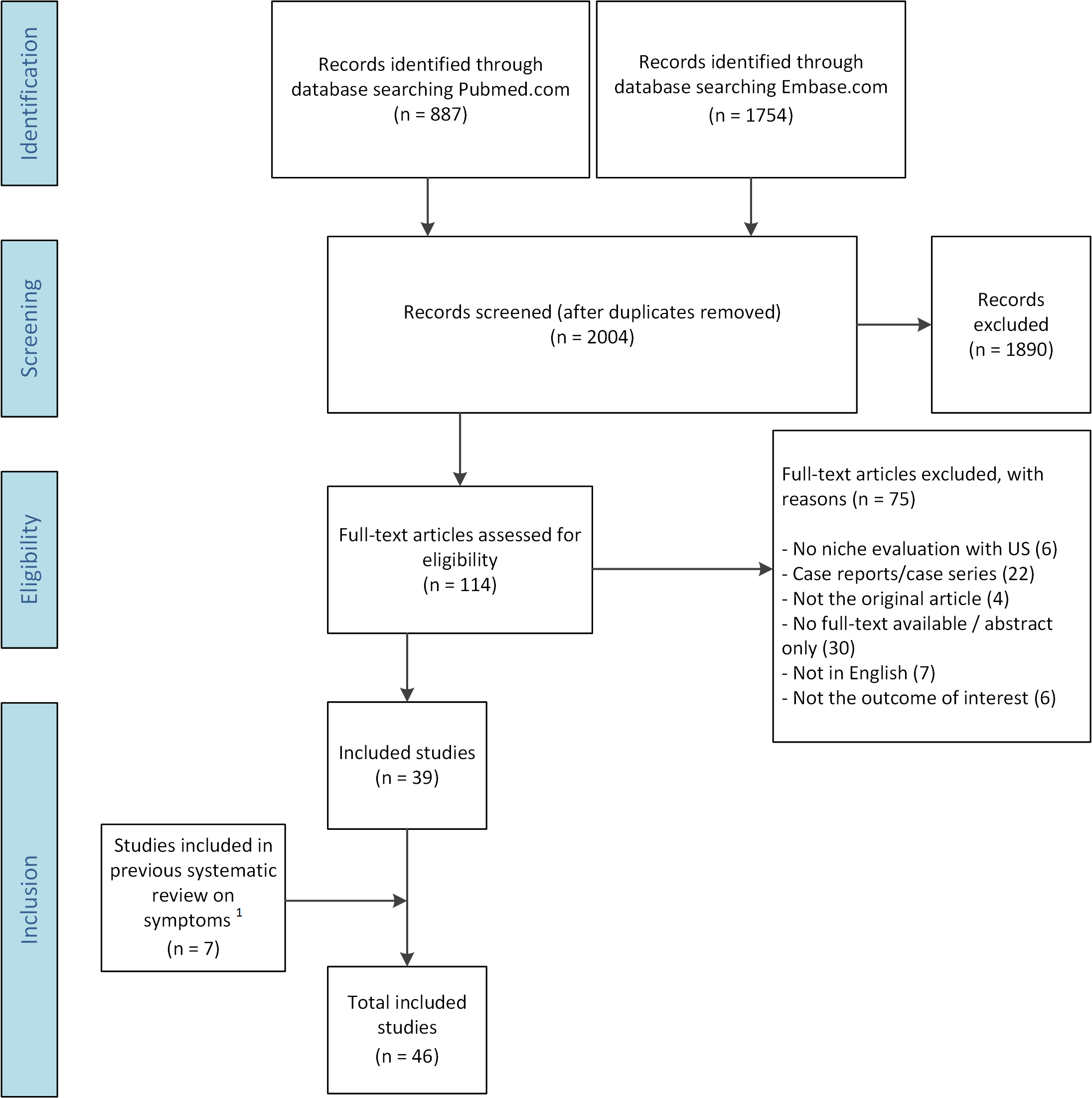

Supplement: Supplementary file 5 — Supplementary material 5 (TIFF 14141 kb) [file 11136_2019_2376_MOESM5_ESM.tif]
