# Supplementary material for: Niche-related outcomes after caesarean section and quality of life: a focus group study and review of literature
Source: Qual Life Res. 2019 Dec 16;29(4):1013–25. doi: 10.1007/s11136-019-02376-6 (PMC7142042; doi:10.1007/s11136-019-02376-6)
Supplement: Supplementary file 6 — Supplementary material 6 (DOCX 33 kb) [file 11136_2019_2376_MOESM6_ESM.docx]

Electronic Supplementary material – Online Resource 6

Scroll down for complete table.

We extracted data from the separate articles and divided the table according to population and outcomes. In summary, eleven studies reported on gynaecological symptoms without evaluating therapy and 24 studies reported on the effect of therapy (hormonal or surgical) on niche-related gynaecological symptoms. Eight and four studies reported on fertility and obstetrics related outcomes after niche therapy, respectively. Twelve studies reported on obstetric outcomes in relation to a sonographic identified niche or thin myometrium without intervention.

| ***Leading author*** | ***Design*** | ***N*** | ***Population*** | ***Intervention(s)*** | ***Outcome*** |
| --- | --- | --- | --- | --- | --- |
| **Gynaecological symptoms associated with the presence of a niche** | | | | | |
| *No selection, random sample of women with a history of CS:* | | | | | |
| *Bij de Vaate, 2011* | *Prospective* | *225* | *Women 6-12 months after a CS* | *NA* | *Postmenstrual spotting* |
| Van der Voet*,* 2014 | Prospective | 263 | Random sample of patients after CS | NA | Postmenstrual spotting, urinary incontinence |
| *Menada Valenzano, 2006* | *Case-control* | *217* | *Vaginal birth (n=101) or CS (n=116)* | *NA* | *AUB or postmenstrual spotting* |
| Au, 2016 | Retrospective | 183 | Patients with at least one previous CS with request of early TOP (6-8 weeks pregnancy) | NA | Successful TOP defined as 'absence of intrauterine gestational sac at TVUS, without surgical intervention' |
| *Selected population consisting of women with a niche, symptomatic or asymptomatic:* | | | | | |
| Wu, 2018 | Retrospective | 121 | Patients with a niche confirmed on TVUS | NA | Postmenstrual spotting |
| *Selected population consisting of symptomatic women with a niche:* | | | | | |
| *Thurmond, 1999* | *Prospective* | *310* | *Women with abnormal uterine bleeding, evaluation with SHG, independent of obstetric history* | *NA* | *Postmenstrual spotting* |
| *Monteagudo, 2001* | *Prospective* | *44* | *Women with a history of CS, who underwent SHG for various gynaecologic indications* | *NA* | *AUB* |
| *Uppal, 2011* | *Prospective* | *71* | *Women referred for gynaecological ultrasound* | *NA* | *AUB (>7 days and/or spotting after period)* |
| *Fabres, 2003* | *Retrospective* | *92* | *Women with history of CS and a niche on TVUS, assessed for other gynaecological reasons* | *NA* | *Postmenstrual spotting or midcycle blood loss* |
| *Wang, 2009* | *Cross-sectional* | *207* | *Women with a history of CS and a niche on TVUS, assessed for other gynaecological indications* | *NA* | *Postmenstrual spotting, dysmenorrhea, chronic pelvic pain, dyspareunia* |
| Tsuji, 2015† | Survey | 189 | Patients with niche and secondary infertility after CS | NA | Prolonged menstruation, abnormal uterine bleeding in follicular/ovulatory/luteal phase, dysmenorrhea, chronic pelvic pain |
| **Gynaecological symptoms in patients treated for niche-related symptoms** | | | | | |
| Vervoort, 2018 | RCT | 95 | Patients with previous CS that present with postmenstrual spotting, niche with RMT ≥3mm | Hysteroscopic niche resection vs expectant management | Postmenstrual spotting, dysmenorrhea, pain during micturition, QoL, sexual functioning |
| Raimondo, 2015 | Prospective | 120 | Symptomatic patients with a niche | Hysteroscopic niche resection ('isthmoplasty') | Postmenstrual abnormal uterine bleeding, suprapubic pelvic pain |
| Zhang, 2016 | Prospective | 142 | Patients treated for caesarean scar defect | 1) laparoscopy, 2) vaginally, 3) hysteroscopy, 4) oral contraceptives, 5) LNG-IUD | Duration of menstruation |
| Vervoort, 2018 | Prospective | 101 | Women with large niche with postmenstrual spotting/dysmenorrhea/intrauterine fluid accumulation/ or difficulties with ET | Laparoscopic niche resection | Postmenstrual spotting days, dysmenorrhea, QoL |
| Muzii, 2017 | Case-control | 47 | Women with niche and postmenstrual AUB, without (23, intervention) and with (24, expectant) wish to conceive | Hysteroscopic niche resection vs expectant management | Postmenstrual abnormal uterine bleeding |
| Wang, 2011 | Retrospective | 57 | Patients with AUB and niche | Hysteroscopic niche resection | Duration of menstruation |
| Chen, 2014 | Retrospective | 64 | Women receiving transvaginal intervention for niche, at least 1 CS | Transvaginal surgery | Prolonged menstrual bleeding |
| Schepker, 2015 | Retrospective | 13 | Presentation with bleeding abnormality, pain or secondary subfertility, at least one previous CS | Laparotomic niche resection | Bleeding abnormalities, pain |
| Li, 2016 | Retrospective | 40 | Women with a niche | Scar defect repair by combination laparoscopy/hysteroscopy | Duration of menstruation, abdominal pain |
| Yang, 2016 | Retrospective | 25 | Women with a niche | Hysteroscopic electrotomy | Duration of menstruation |
| Zhang, 2016 | Retrospective | 124 | Patients with niche and prolonged menstruation (13-20 days) | Transvaginal vs laparoscopic niche repair | Prolonged menstrual bleeding |
| Zhou, 2016 | Retrospective | 121 | Patients with history of CS, presented with postmenstrual spotting | Vaginal niche repair | Postmenstrual spotting, pelvic pain, dyspareunia |
| Cohen, 2017† | Retrospective | 8 | Patients with previous hysteroscopic niche resection (recurrence of symptoms) | Second hysteroscopic niche resection | Days with blood loss per cycle |
| Wu, 2017 | Retrospective | 145 | History of CS, defect on ultrasound, irregular vaginal bleeding and/or abdominal pain | Laparoscopy/hysteroscopy vs hormonal treatment | Menstruation duration, abdominal pain, QoL |
| Xu, 2017 | Retrospective | 123 | At least one previous CS, prolonged menstrual period >7 days | Laparoscopic or vaginal niche repair | Duration of menstruation |
| Zhang, 2017 | Retrospective | 146 | Symptomatic patients with a niche | Laparoscopic niche resection | Duration of menstruation |
| Zhou, 2017 | Retrospective | 124 | At least one previous CS, postmenstrual spotting and niche present | Vaginal niche repair | Duration of menstruation |
| Cheng, 2018* | Retrospective | 106 | Women with a niche | Surgical niche treatment (hysteroscopy, laparoscopy, vaginal) vs no treatment | Prolonged menstrual bleeding > 7 days, abdominal pain, dyspareunia, dysmenorrhea |
| Lv, 2018* | Retrospective | 82 | Patients with a symptomatic niche | Laparoscopic niche resection under hysteroscopic guidance vs hysteroscopic surgery | Duration of menstruation |
| Zhou, 2018* | Retrospective | 51 | Patients with symptoms and niche or RMT < 3 mm | Vaginal niche repair | Duration of menstruation |
| Chen, 2019 | Retrospective | 241 | Patients with AUB and niche | Transvaginal niche repair | Duration of menstruation |
| Li, 2019 | Retrospective | 52 | Patients with niche | Laparoscopic niche resection under hysteroscopic guidance vs hysteroscopic surgery | Duration of menstruation (cure, improvement, invalid) |
| Shapira, 2019* | Retrospective | 67 | Patients with a symptomatic niche | Hysteroscopic niche resection | Postmenstrual bleeding, pelvic pain, infertility |
| Zhang, 2019* | Retrospective | 67 | Patients with a symptomatic niche | Transvaginal niche repair vs laparoscopic repair | Duration of menstruation |
| **Reproductive outcomes associated with the presence of a niche or thin lower uterine segment – with or without treatment** | | | | | |
| *Fertility related outcomes:* | | | | | |
| Schepker, 2015 | Retrospective | 13 | Presentation with bleeding abnormality, pain or secondary subfertility, at least one previous CS | Laparotomic niche resection | Secondary subfertility |
| Cohen, 2017† | Retrospective | 8 | Patients with previous hysteroscopic niche resection (recurrence of symptoms) | Second hysteroscopic niche resection | Wish to conceive, conception, miscarriage, live birth |
| Zhang, 2017 | Retrospective | 146 | Symptomatic patients with a niche | Laparoscopic niche resection | Wish to conceive, pregnancy rate, miscarriage rate, caesarean scar pregnancy |
| Cheng, 2018* | Retrospective | 106 | Women with a niche | Surgical niche treatment (hysteroscopy, laparoscopy, vaginal) vs no treatment | Characteristics of subsequent pregnancy: secondary subfertility, abortion, caesarean scar pregnancy |
| Lv, 2018* | Retrospective | 82 | Patients with a symptomatic niche | Laparoscopic niche resection under hysteroscopic guidance vs hysteroscopic surgery | Pregnancy and miscarriage rate |
| Zhou, 2018* | Retrospective | 51 | Patients with symptoms and niche or RMT < 3 mm | Vaginal niche repair | Pregnancy and miscarriage rate |
| Shapira, 2019* | Retrospective | 67 | Patients with a symptomatic niche | Hysteroscopic niche resection | Spontaneous pregnancy and live birth rate |
| Zhang, 2019* | Retrospective | 67 | Patients with a symptomatic niche | Transvaginal niche repair vs laparoscopic repair | Pregnancy and miscarriage rate |
| Tsuji, 2015† | Survey | 189 | Patients with niche and secondary infertility after CS | OI, IUI, IVF-ET versus drainage of fluid versus surgical treatment | Pregnancy rate |
| *Obstetrics related outcomes:* | | | | | |
| Sanlorenzo, 2013 | Prospective | 214 | Women with one previous CS undergoing repeat CS | NA | Intraoperative condition of LUS |
| Pomorski, 2014 | Prospective | 41 | Patients with history of CS referred for repeat CS | NA | Intraoperative appearance of LUS |
| Kinjo, 2015 | Prospective | 89 | Women with and without previous CS, planned for elective CS | NA | Risk of uterine scar defect |
| Singh, 2015 | Prospective | 142 | Patients with one previous CS undergoing TOLAC | NA | Successful VBAC |
| Uharček, 2015 | Prospective | 336 | Patients with one previous CS undergoing repeat CS | NA | Intraoperative appearence of LUS |
| Barzilay, 2017 | Prospective | 57 | Women in active labour with and without previous CS | NA | Successful VBAC |
| Ionescu, 2017 | Prospective | 275 | Repeat CS after one previous CS | NA | Risk of uterine rupture |
| Baranov, 2018 | Prospective | 80 | Women with one previous CS > 37 weeks | NA | Successful VBAC |
| Al-Nakkash, 2019 | Prospective | 300 | Pregnant women with ≥ 2 previous CSs | NA | Scar dehiscence at repeat CS in relation to preoperative TAUS |
| Tazion, 2019 | Prospective | 70 | Pregnant women with ≥ 1 previous CSs | NA | Scar dehiscence / rupture at repeat CS in relation to preoperative TAUS |
| Sharma, 2015 | Case-control | 100 | Women with (group 1) and without (group 2) previous CS, planned for elective CS | NA | Risk of uterine dehiscence/rupture |
| Cohen, 2017 | Retrospective | 8 | Patients with previous hysteroscopic niche resection (recurrence of symptoms) | Second hysteroscopic niche resection | Risk on abnormal adhesive placenta |
| Cheng, 2018* | Retrospective | 106 | Pregnant women with a niche | Surgical niche treatment (hysteroscopy, laparoscopy, vaginal) vs no treatment | Development of placenta previa/accreta Uterine dehiscence or rupture, mode of delivery |
| Lv, 2018* | Retrospective | 82 | Patients with a symptomatic niche followed until subsequent pregnancy and delivery | Laparoscopic niche resection under hysteroscopic guidance vs hysteroscopic surgery | Mode and timing of delivery, uterine rupture |
| Zhou, 2018* | Retrospective | 51 | Patients with symptoms and niche or RMT < 3 mm | Vaginal niche repair | Risk of uterine rupture, neonatal outcomes |
| Shapira, 2019* | Retrospective | 67 | Patients with a symptomatic niche followed until subsequent pregnancy and delivery | Hysteroscopic niche resection | Niche pregnancy, placenta accrete, uterine rupture |
| Zhang, 2019* | Retrospective | 67 | Patients with a symptomatic niche followed until subsequent pregnancy and delivery | Transvaginal niche repair vs laparoscopic repair | Mode of delivery |

**Online Resource 6.** Overview of published literature on presence of a niche or thin lower uterine segment (on ultrasound) after CS and the association with symptoms or problems, with or without applied therapy for these symptoms or problems.

In *Italic:* studies presented in table S2 by Bij de Vaate *et al.* *(Ultrasound Obstet Gynecol. 2014 Apr;43(4):372-382)*

CS, Caesarean section; AUB, abnormal uterine bleeding; LUS, lower uterine segment; TOP, termination of pregnancy; TVUS, transvaginal ultrasound; SHG, sonohysterography; RCT, randomized controlled trial; RMT, residual myometrium thickness; QoL, Quality of Life; ET, embryo transfer; LNG-IUD, levonorgestrel releasing intra-uterine device; ART, assisted reproductive technology; OI, ovulation induction; IUI, intra uterine insemination; IVF-ET, in vitro fertilization embryo transfer; TOLAC, trial of labour after caesarean; VBAC, vaginal birth after caesarean; TAUS, transabdominal ultrasound; NA, not applicable. † Studies included both in relation to gynaecological and fertility related outcomes. * Studies included in relation to gynaecological, fertility and obstetrics related outcomes.
